# COVID-19’s disruptions to cancer care pathways and widening of health inequalities in the UK: a systematic review

**DOI:** 10.1186/s12913-026-14313-8

**Published:** 2026-03-26

**Authors:** Cheuk Him Michael Lam, Kar Chun Cheung, Thomas Mason, Bruce Hollingsworth

**Affiliations:** 1 Division of Health Research, Faculty of Health and Medicine, Lancaster University, Lancaster, UK; 2 Jockey Club School of Public Health and Primary Care, The Chinese University of Hong Kong, Hong Kong, China; 3 Biomedical Engineering Services Section, Hospital Authority, Hong Kong, China

**Keywords:** COVID-19, Cancer care, Health inequality, Socioeconomic status, Ethnicity, Healthcare access, Health policy, Systematic review

## Abstract

**Background:**

The COVID-19 pandemic has significantly impacted cancer care services in the United Kingdom (UK), potentially exacerbating pre-existing health inequalities. While emerging studies have documented service disruptions, a comprehensive synthesis of how these disruptions have widened disparities remains absent. This systematic review examines the extent to which the pandemic disrupted the cancer care pathway and intensified existing disparities across the UK, identifying key sociodemographic and geographical factors influencing access to services.

**Methods:**

A systematic search of PubMed, Scopus, and CINAHL was conducted for studies published between January 2020 and October 2024. Eligible studies included observational and empirical research examining disparities in cancer screening, diagnosis, treatment, and outcomes during the COVID-19 pandemic, as well as the corresponding mitigation strategies. Data extraction followed a structured approach using a custom-developed extraction form designed for this review. Study quality was appraised using a bespoke scoring system, classifying studies as high, moderate, or low importance. Narrative synthesis, following the framework outlined by Popay et al., was then employed to identify key themes and explore relationships between findings.

**Results:**

30 out of 457 studies met the inclusion criteria. The review found that socioeconomic status (SES) emerged as the most significant determinant, with individuals from deprived areas experiencing greater barriers to screening, urgent referrals, and treatment access, leading to poorer patient outcomes. Ethnic minorities, particularly Black patients, faced disproportionate reductions in hospital admissions and cancer screening participation. Age-related disparities were also evident, as older adults maintained higher screening rates but faced greater COVID-19 risks, while younger adults from lower-income backgrounds encountered delays in diagnosis and treatment.

**Conclusions:**

The review highlights that the COVID-19 pandemic has exacerbated existing inequalities in UK cancer care, with SES, ethnicity, and age emerging as key determinants. Targeted interventions are essential, including the establishment of COVID-free “cold sites”, deployment of mobile screening units, and culturally tailored outreach programmes for ethnic minority communities. Strengthening regional healthcare capacity and conducting longitudinal assessments will be crucial in addressing disparities and ensuring equitable cancer care. Future research should focus on the long-term consequences of these disruptions on cancer outcomes and healthcare resilience.

**Systematic review registration:**

The protocol for this systematic review was registered on PROSPERO under the ID CRD42024602280.

**Supplementary Information:**

The online version contains supplementary material available at 10.1186/s12913-026-14313-8.

## Background

The COVID-19 pandemic caused widespread disruption to cancer care services across the United Kingdom (UK), with significant implications for long-standing health inequalities. Substantial interruptions to screening, diagnosis, and treatment services have raised concerns about their long-term impact on patient outcomes [1]. During the first wave, urgent referrals from primary care for suspected cancer fell by 56% compared to pre-pandemic levels [2]. Even brief delays in treatment can have serious consequences; a one-month delay has been associated with a 6% to 13% increase in mortality risk [3]. Modelling studies estimate that diagnostic delays alone could lead to up to 3,621 excess deaths from breast, colorectal, oesophageal, and lung cancers in England over five years, equating to 63,229 additional years of life lost (YLL) [4].

In this systematic review, the terms “inequality” and “disparity” are used interchangeably to denote measurable differences in cancer outcomes or access between population groups, following common usage in health services research [5]. Even before the pandemic, pre-existing inequalities in cancer care were evident across the continuum – from disease burden and detection, through access to appropriate treatment, to outcomes – with disparities operating differently at each stage of the care pathway [6]. For instance, incidence rates for all cancers combined were 17% higher among men and 11% higher among women in the most deprived areas compared to the least deprived [7], while age-standardised cancer incidence rates for women ranged from 524 per 100,000 in London to 603 in the North East in 2019 [8]. Screening participation, on the other hand, was lower in deprived communities, with breast cancer screening uptake at 49% in the most deprived areas versus 71% in the least deprived [9]. Access to timely referrals and diagnostics also varied notably by socioeconomic status (SES), ethnicity, and geography [10, 11]. Regarding quality and outcome of treatment, patients from deprived communities were also less likely to receive breast-conserving surgery and experience higher mortality rates [12]. Moreover, longer travel times to healthcare facilities are associated with poorer cancer survival, particularly in deprived regions where reliance on public transport is greater [13].

These disparities do not only influence individual outcomes but also impose a considerable burden on the healthcare system and society. Socioeconomic inequality accounts for roughly 1 in 3 premature deaths [14], marking it a critical public health issue in the UK. As highlighted by the Institute for Fiscal Studies’ Deaton Review, health inequalities in the country are shaped by a complex web of social, economic, and environmental factors that extend beyond traditional deprivation indices [15]. These include early-life disadvantage, cumulative exposure to risk, and divergent life trajectories, all of which contribute to stark regional differences in health. With the onset of the COVID-19 pandemic, this already complex landscape of health inequalities has been further complicated, potentially exacerbating existing disparities and introducing new challenges in cancer care provision across the UK. While some studies have explored socioeconomic influences on COVID-19 outcomes among cancer patients [11], there remains a lack of comprehensive, nation-wide summary of these impacts. This gap is especially important given that health inequalities significantly shaped cancer care outcomes even prior to the pandemic. Variations in healthcare infrastructure, population density, and deprivation levels likely contributed to uneven service disruptions. The rapid shift to telemedicine and altered screening protocols may have further widened access disparities. Notably, even well-intentioned interventions aimed at improving health outcomes can sometimes have unintended consequences. For example, the National Health Service (NHS) Diabetes Prevention Programme has in some cases reinforced disparities, with lower engagement among deprived and minority populations, partly due to its reliance on routine primary care referrals [16].

This review addresses these gaps by examining how the pandemic affected cancer care disparities across the UK. It explores which population groups and regions were most affected, identifies key sociodemographic and geographical factors influencing disparities, and evaluates the mitigation strategies adopted during the pandemic and their relative effectiveness. In doing so, the review aims to inform equitable service recovery, strengthen healthcare resilience, and support the development of inclusive, evidence-based policy responses – an objective aligned with the NHS Constitution’s legally mandated commitment to reduce inequalities in access and outcomes [17], and the National Institute for Health and Care Research’s (NIHR) strategic priority to fund research that addresses the needs of underserved populations and reduces health disparities [18].

## Methods

The protocol for this review was registered with PROSPERO under Registration Number: CRD42024602280. This systematic review aimed to synthesise quantitative evidence on how the COVID-19 pandemic impacted disparities in cancer care across the UK, with a focus on identifying key sociodemographic and geographical factors contributing to unequal access and outcomes.

The review was conducted in accordance with a pre-specified protocol to ensure transparency, reproducibility, and methodological rigour, particularly drawing from the guidance developed by the Cochrane Handbook for Systematic Reviews of Interventions [19]. The Population, Intervention, Comparison, and Outcomes (PICO) framework was used to guide the formulation of the research question and inclusion criteria [20]:

- Population: Cancer patients within the UK (including England, Scotland, Wales, and Northern Ireland).
- Intervention: Impact of the COVID-19 pandemic on cancer care services.
- Comparison: Cancer care provision and treatment outcomes before and after the onset of the pandemic.
- Outcomes: Performance metrics for cancer care provision and quality, including disparities across demographic, socioeconomic, and geographical groups.

A narrative synthesis approach was employed to integrate evidence from studies with diverse designs and metrics. This approach was guided by established guidelines for narrative synthesis in systematic reviews developed by Popay et al. [21], which is particularly suited for examining complex public health interventions across heterogeneous contexts like those in this study. The narrative synthesis allowed for the exploration of relationships within and between studies, consideration of contextual factors, and identification of recurring patterns in the data. To classify and interpret reported performance measures, the review applied Donabedian’s model of healthcare quality [22], which organises indicators into three categories:

- Structure: Characteristics of the healthcare setting (e.g., availability of facilities, workforce capacity, infrastructure).
- Process: Activities involved in healthcare delivery (e.g., referral pathways, diagnostic procedures, treatment initiation).
- Outcome: Effects of care on patients (e.g., survival rates, quality of life, delays, patient satisfaction).

### Search strategy

A comprehensive search strategy was developed to identify studies examining the impact of the COVID-19 pandemic on cancer care disparities across the UK. Three databases were searched to ensure comprehensive coverage of the medical, health services, and social sciences literature: PubMed, Scopus, and CINAHL. These databases were selected for their complementary strengths, with PubMed offering focused biomedical coverage, Scopus providing extensive interdisciplinary indexing, and CINAHL including nursing and allied health research relevant to service delivery and access.

The search strategy combined controlled vocabulary, such as Medical Subject Headings (MeSH) in PubMed, and free-text terms. These terms were structured around five core concepts: cancer, COVID-19, healthcare access and outcomes, health disparities, and the UK context. Within each concept, multiple synonyms and related terms were used to ensure sensitivity. For example, cancer-related terms included “neoplasms”, “oncology”, “malignancy”, “tumour”, and “carcinoma”, while disparity-related terms included “inequality”, “socioeconomic”, “ethnicity”, and “geographical”. Boolean operators were used to combine concepts, and truncation was applied to capture variations in terminology. The syntax was tailored to the indexing requirements of each database. As an example the PubMed search is specified below, the full search terms for each database and the resulting number of papers can be found in Additional File 1, copies of all database searches can be obtained by contacting the corresponding author:

(Neoplasms[MeSH] OR cancer*[Title/Abstract] OR oncolog*[Title/Abstract] OR malignan*[Title/Abstract]) AND (COVID-19[MeSH] OR SARS-CoV-2[MeSH] OR coronavirus[Title/Abstract] OR pandemic[Title/Abstract]) AND (inequalit* OR inequit* OR disparit* OR equity OR equitable OR socioeconomic OR access* OR “Healthcare Disparities”[MeSH]) AND (“United Kingdom”[MeSH] OR UK[Title/Abstract] OR Britain[Title/Abstract] OR England[Title/Abstract] OR Scotland[Title/Abstract] OR Wales[Title/Abstract] OR NHS[Title/Abstract]) AND (“2020/01/01”[Date - Publication]: “3000”[Date - Publication])

### Inclusion and exclusion criteria

Studies were eligible for inclusion if they presented empirical data on cancer care within the UK and examined the impact of the COVID-19 pandemic on service delivery or outcomes. Eligible studies were required to focus on cancer patients and report on disruptions to screening, diagnosis, treatment, or follow-up care during the pandemic, with a particular emphasis on disparities across sociodemographic or geographical groups. Studies had to include stratified findings by factors such as SES, ethnicity, age, gender, or region to enable meaningful analysis of inequalities. Only studies published between January 2020 and October 2024 were considered, encompassing the onset and evolution of the pandemic. Observational study designs were included, such as retrospective and prospective cohorts, cross-sectional studies, quasi-experimental designs, and time-series analyses. Modelling studies were eligible where they estimated pandemic-related impacts on cancer care and stratified findings by relevant population characteristics. Studies had to present quantitative data, with outcomes that could be assessed in relation to disparities in access or provision. Studies were excluded if they did not focus specifically on cancer patients, lacked UK-specific data, or failed to provide stratified analysis related to sociodemographic or regional disparities. These criteria ensured the review captured robust evidence on how the pandemic influenced disparities in cancer care across different population groups and service settings. Full list of included papers can be found in Additional File 2.

### Data extraction

Data was extracted using a custom-developed extraction form tailored to this review. This form was designed to ensure systematic and comprehensive capture of relevant data across studies. This is consistent with standard approaches to narrative synthesis, where review-specific data extraction frameworks are often employed in similar studies. Two reviewers independently performed the data extraction, and any discrepancies were resolved through discussion to ensure consistency and accuracy. The extracted information included key study characteristics such as authorship, year of publication, journal, study design, sampling method, sample size, and duration of study. Population-level data were recorded, covering cancer types studied, age and gender distributions, ethnicity profiles, and socioeconomic indicators, including the use of the Index of Multiple Deprivation (IMD) where applicable. The geographical context was also documented, including specific regions within the UK, urban or rural classifications, and any reference to healthcare system structures, such as the involvement of specific NHS trusts. COVID-19-related information was captured in detail. This included the specific pandemic timeframes under study, any reference to lockdown periods or public health policy changes affecting service delivery, and the stages of the cancer care continuum examined, such as screening, diagnosis, treatment modalities (e.g. surgery, chemotherapy, radiotherapy), and follow-up care. Key findings were recorded, including reported effect sizes, confidence intervals, statistical analyses performed, and the authors’ main conclusions. Additional data were collected on the extent to which studies considered potential confounding factors and the methods used to address them. Where applicable, details of interventions implemented to mitigate disparities were documented, alongside their duration and intensity. Information on study limitations, both as identified by the authors and as assessed during extraction, was also included to inform critical appraisal. The extraction form can be found in Additional File 3.

### Risk of bias and quality assessment

A rigorous appraisal process was implemented to assess the quality, relevance, and potential for bias in the studies included in this review. A bespoke scoring system was developed and applied, tailored to the objectives of the review and the heterogeneity of the evidence base. The use of a custom approach is justified and consistent with Popay et al.’s observation that narrative synthesis “does not rest on an authoritative body of knowledge” and requires reviewers to “combine sound methodology with creative interpretative work” when appraising diverse evidence [21]. This aligns with common practice in narrative syntheses, where flexible, review-specific appraisal frameworks are frequently employed. The appraisal framework comprised five domains: methodological rigour, relevance to the research question, scope of cancer care coverage, sample coverage, and the significance of effect size. These domains were selected because they reflect established principles for assessing observational and service-evaluation evidence, particularly the need to consider internal validity, applicability, and the extent to which studies contribute meaningfully to answering the review question. Each study was independently scored across these domains, with final classifications as high, moderate, or low importance based on their total score:

- Methodological rigour encompassed an evaluation of study design and the extent to which bias was minimised. This included considerations such as sample representativeness, control for confounding variables, and appropriateness of analytic methods.
- Relevance was determined by how directly the study addressed disparities in cancer care during the COVID-19 pandemic within the UK context.
- Scope of cancer care coverage assessed whether studies provided insights into multiple stages of the cancer care continuum, including screening, diagnosis, treatment, and follow-up services.
- Sample coverage was used to assess geographical and population-level representativeness, with higher scores assigned to studies drawing on UK-wide data or incorporating diverse demographic groups.
- Effect size and statistical significance were judged based on reported estimates such as odds ratios, relative risks, or confidence intervals, and the strength of associations between pandemic-related disruptions and disparities.

Risk of bias was explicitly considered within the methodological rigour domain. Studies were appraised for potential selection bias, confounding, and issues related to data completeness or transparency in reporting. This multi-domain structure enhances rigour by formalising how quality, relevance, and contribution are judged, imposing consistent standards across heterogeneous evidence, and ensuring that scoring decisions can be independently replicated.

To identify the most influential sociodemographic and geographical factors associated with disparities, a weighted composite score was generated for each factor. This combined three components: the frequency with which the factor was identified in high or moderate importance studies, the degree of consistency across findings, and the overall magnitude of its reported effect. This approach enabled the prioritisation of the most robustly supported factors for inclusion in the subsequent narrative synthesis. The appraisal form and scoring system used can be found in Additional File 4.

### Data synthesis

Given the methodological diversity of the included studies, a narrative synthesis approach was employed to integrate findings and generate a coherent understanding of how the COVID-19 pandemic exacerbated disparities in cancer care across the UK. A meta-analysis was not feasible due to the heterogeneity in study designs, outcome measures, and populations studied. The narrative method allowed for the systematic exploration of relationships between study findings, while accounting for context-specific variations in cancer care delivery and population characteristics. The synthesis followed the framework proposed by Popay et al. [21], which is widely used in public health and health services research to guide transparent and theory-informed narrative reviews. This framework consists of four interrelated elements. First, a preliminary synthesis was developed to describe and organise the data, identifying patterns across studies in terms of cancer care disruptions and the populations affected. Second, a conceptual framework was applied to examine how the pandemic affected the organisation and delivery of cancer care, drawing on Donabedian’s model of healthcare quality [22] to guide the categorisation of disruptions across structural, procedural, and outcome domains. Third, relationships were explored, including how disparities varied by sociodemographic and geographical factors, and the stage of care. Finally, the robustness of the synthesis was assessed by considering the quality and importance of the underlying evidence, giving greater weight to findings from studies categorised as high or moderate importance. This structured approach enabled the review to identify key sociodemographic and geographical factors influencing disparities, as well as to highlight gaps in the literature. It also allowed for the integration of diverse study designs and outcome metrics in a way that maintained analytical depth while preserving contextual nuance.

## Results

### Study selection

**Fig. 1:**
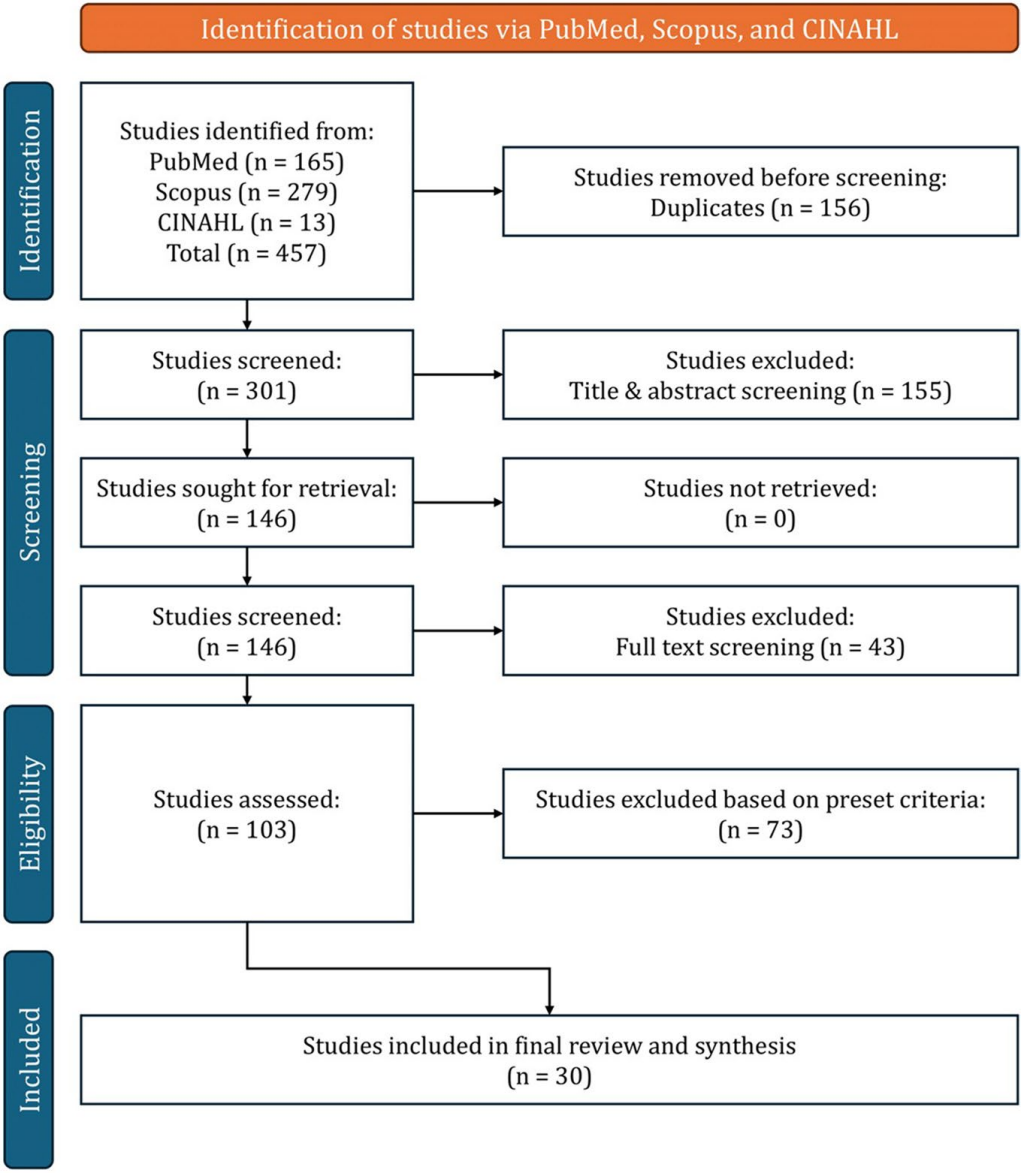
PRISMA flow diagram for the research studies search

The initial search using the predetermined search terms yielded a total of 457 papers. Following a rigorous screening process involving examination of titles, abstracts and full texts, and application of the eligibility criteria, 30 papers were ultimately selected for inclusion in the review. Figure 1 below provides a visual summary of the literature search and sift process.

### Study characteristic and quality

The majority of included studies (*n* = 22) were observational in nature, encompassing retrospective cohort studies, cross-sectional analyses, surveys, and interrupted time series designs. These studies offered valuable insight into the real-world impacts of the COVID-19 pandemic on cancer care delivery and outcomes across different population groups. Notable examples included a national population-based analysis of colorectal cancer (CRC) care in England [23], and a large-scale interrupted time series study covering populations in England, Scotland, and Wales [24]. A smaller number of studies (*n* = 3) employed modelling techniques to project the long-term consequences of pandemic-related service disruptions. One study modelled the effects of diagnostic delays on CRC survival [25], while another used microsimulation to estimate the impact of screening pauses on CRC mortality [26]. In addition to empirical studies, the review included several policy-focused papers and commentary articles (*n* = 5), which provided broader contextual analysis of the structural and system-level implications of the pandemic for cancer care in the UK [27, 28]. Although all studies focused on the UK, the geographical coverage varied. 15 studies provided UK-wide analyses, while others concentrated on specific nations: England (*n* = 11), Scotland (*n* = 1), Wales (*n* = 2), and Northern Ireland (*n* = 1). A small number of studies (*n* = 2) compared findings across multiple UK nations. Cancer type also varied, with some studies investigating all cancers collectively (*n* = 12), and others focusing on specific cancers, including colorectal (*n* = 7), breast (*n* = 5), and other tumour types. Sample sizes ranged considerably, from studies involving a few hundred individuals to nationwide datasets covering millions. The largest study included data from approximately 32 million people across the UK [24]. Most studies examined multiple sociodemographic characteristics, with age (*n* = 25), SES (*n* = 18), gender (*n* = 17), and ethnicity (*n* = 14) being the most frequently analysed factors. SES was typically measured using the IMD. Many studies also incorporated geographical variables to explore regional variations in cancer care access or outcomes. The timeframes covered generally spanned from the onset of the pandemic in early 2020 through to 2021 or 2022. Several studies included comparisons with pre-pandemic baselines to assess changes in service utilisation, access, or patient outcomes over time.

The overall quality of the included studies varied. 7 studies were classified as high importance, demonstrating strong methodological rigour, robust sample coverage, and clearly reported effect estimates. 19 studies were rated as moderate importance, typically limited by partial coverage of the cancer care continuum, narrower geographical scope, or incomplete adjustment for confounders. 4 studies were deemed low importance due to methodological limitations, such as small sample sizes, limited generalisability, or insufficient reporting of outcomes and statistical significance. Nonetheless, the majority of the evidence base was of moderate to high quality, offering a reliable foundation for synthesis and interpretation.

Please refer to Table 1 for a comprehensive overview of the key characteristics of each included study, including cancer types, study design, population, key factors examined, and main findings.

**Table 1:** Summary of key characteristics of included studies

Author, year	Cancer type(s) studied	Study design	Population	Key sociodemographic factors examined	Key geographic region examined	Key findings (how the factors impacted cancer care)	Importance / Score
Aggarwal et al., 2024 [27]	All cancers	Policy Review	N/A (review of national data and policies)	Age, gender, ethnicity, SES	Entire UK, with some discussion of differences between England, Scotland, Wales, and Northern Ireland	More deprived areas have higher cancer incidence and mortality, older age groups face higher survivor rates with comorbidities, racial and ethnic minorities encounter more access barriers, and regions like the North East show higher cancer rates and better screening uptake than others.	Moderate
Ali & Riches, 2021 [29]	All cancers	Literature review	N/A (review of multiple studies)	Age, comorbidities	Primarily focused on UK, with some global context	Elderly patients and those with comorbidities are at higher risk of severe COVID-19 outcomes, cancer patients particularly so. Reduced healthcare capacity led to a significant drop in urgent referrals, while screening disruptions and diagnostic delays further hindered timely cancer detection and treatment.	Moderate
Baxter et al., 2021 [30]	All cancers, with specific data on colorectal, breast, lung, and haematological cancers	Observational study using real-time national data	Nationwide data from NHS Scotland	Age and comorbidities (mentioned as risk factors but not specifically analysed)	3 regional networks in Scotland: North of Scotland Cancer Alliance (NCA), West of Scotland Cancer Network (WoSCAN), and South East Cancer Network (SCAN)	Regions like WoSCAN experienced a greater decrease in patient attendances compared to SCAN and NCA, though the recovery rates were similar. Systemic anticancer therapy delivery decreased across Scotland with regional variations, but no significant link was found between high COVID-19 rates and cancer service provision.	Moderate
Boyle et al., 2021 [31]	CRC	National survey	148 NHS hospitals providing CRC services in England and Wales	Not specifically analysed	21 Cancer Alliance regions in England plus Wales	Hospitals with access to “cold sites” had significantly higher activity levels for key cancer treatments, including diagnostic colonoscopy and various cancer surgeries. There was no significant link between high regional COVID-19 rates and CRC services, but a substantial decrease in CRC service provision occurred across England and Wales during the pandemic’s first peak.	High
Boyle et al., 2024 [23]	CRC	National population-based study using routinely collected data	All CRC patients in NHS England	Age, gender, ethnicity, SES (IMD quintiles)	Entire England	The screening age group (60–74 years) showed better recovery in diagnoses and major resections compared to adjacent age groups. A socioeconomic gradient was observed, with fewer diagnoses and resections in more deprived areas. Gender distribution during the pandemic was 55.3% male and 44.7% female, while ethnicity was predominantly White (87.2%), with lower representation from Mixed, South Asian, Black, and other groups.	High
Bright et al., 2023 [32]	CRC screening	Retrospective cohort study using linked administrative data	265,234 individuals aged 60–74 years in Wales	Age, gender, ethnicity, SES (income deprivation quintiles), education deprivation, clinically extremely vulnerable (CEV) status	Whole of Wales, urban (64.2%) vs. rural (35.8%) classification	Older age groups, particularly those aged 70–74, had higher screening uptake. Females consistently outpaced males in uptake, while Asian and unknown ethnic groups had lower rates compared to Whites. Uptake was also lower in more deprived areas and among CEV individuals. Rural areas had slightly higher uptake, but the difference wasn’t significant after adjustments. Overall, inequalities didn’t significantly worsen post-pandemic, though some groups experienced larger declines.	Moderate
Castanon et al., 2021 [33]	Cervical cancer	Modelling study	N/A (population-level modelling)	Age, human papillomavirus (HPV) vaccination status	England	Women aged 40–49 years are expected to be most impacted in both scenarios, while the impact on women aged 25–34 years is mitigated by HPV vaccination. Vaccinated cohorts, primarily younger women, have lower excess cancer rates.	Moderate
Creavin et al., 2023 [34]	CRC	Cross-sectional study	86,850 patients aged 60–74	Age, gender, ethnicity, SES (IMD), smoking status, body mass index (BMI), alcohol consumption, care home residence	England (Bristol, North Somerset and South Gloucestershire)	Deprivation is strongly associated with declining screening and lack of screening records, with the most deprived having higher odds compared to the least deprived. Black individuals also have higher odds of no screening record compared to White individuals. Care home residents show significantly higher odds of declining screening.	Moderate
De Souza et al., 2023 [35]	Hepatocellular carcinoma (HCC)	Prospective multicentre cohort study	164 patients with current or past HCC	Age, gender, ethnicity	8 UK hospitals and 1 German hospital	Ethnicity, age, and hospital variation show no significant association with delays to treatment (DTT).	Moderate
Dema et al., 2023 [36]	Cervical cancer (screening)	Cross-sectional web survey	2949 eligible participants aged 25–59 years	Age, gender, ethnicity, SES (education level, social grade), sexual identity	England, Wales, Scotland	Younger participants (25–29 years) are more likely to report screening in the past year compared to those aged 45–59 years. Gay or lesbian participants are less likely to report screening, and there is some evidence of lower screening uptake in rural areas compared to urban areas.	Moderate
Green et al., 2023 [37]	All cancers (avoidable hospital admissions)	Observational analysis using linked longitudinal studies	29,276 individual level records	Age, gender, ethnicity, SES (housing tenure, IMD quintiles)	England	Older individuals are more likely to experience healthcare disruption, with a mean age of 59.6 years compared to 49.4 years. Females experience slightly higher disruption rates than males, and White individuals are slightly overrepresented among those experiencing disruption. People from the most deprived areas are more likely to face disruption compared to those from the least deprived areas.	Moderate
Greene et al., 2023 [38]	All pathology-confirmed cancers	Retrospective observational study	Whole populations of Northern Ireland, Scotland, and Wales	Age, gender	Northern Ireland, Scotland, Wales	During the pandemic, there was a significant decrease in cancer incidence across all three countries. The largest reductions were seen in colorectal and prostate cancers. Incidence rates partially recovered by the end of 2020 but remained below expected levels. The impact varied by cancer type, country, and time period. Older age groups showed larger decreases in cancer diagnoses.	Moderate
Hamilton et al., 2021 [39]	All cancers (excluding non-melanoma skin cancer)	Population-based observational study	All cancer diagnoses in Northern Ireland	Age, gender	Northern Ireland (entire region)	Overall cancer diagnoses decreased during the pandemic, with the 50–59 age group most affected. Males and younger/middle-aged adults lagged behind in returning to expected diagnosis numbers. Different cancer types showed varying rates of diagnostic recovery.	High
Hull et al., 2021 [40]	Not specified (all non-emergency referrals)	Retrospective cohort study	246,409 referrals in East London	Age, gender, ethnicity, long-term conditions, IMD	East London	Older age groups, South Asian and other ethnic groups had lower odds of referral. Those with 3 + long-term conditions had slightly higher odds.	Moderate
Jiwa et al., 2021 [41]	N/A (breast health screening)	Cross-sectional questionnaire study	3178 respondents across UK	Age, ethnicity, education level	UK-wide	High engagement from Asian/Asian British respondents. 84.6% of respondents aged 21–40.	Low
Kontopantelis et al., 2022 [42]	All cancers (as part of broader mortality study)	Registry-based study using interrupted time-series analysis	3,265,937 deaths in England and Wales	Age, gender, IMD quintiles	10 regions in England and Wales	Strong socioeconomic gradient in excess years of YLL. Males and most deprived areas had higher excess YLL.	High
Lee et al., 2021 [11]	All incident cancers	Case-cohort study	18,917 incident cancer patients from UK Biobank	Age, gender, ethnicity, Townsend Deprivation Index, employment status	UK-wide	Most deprived areas, Black ethnicity, unemployment, and higher BMI associated with increased risk of COVID-19 positivity among cancer patients.	Moderate
Loveday et al., 2021 [25]	CRC	Modelling study	Based on 11,266 CRC cases diagnosed per year via 2 week wait pathway	Age	England	Significant impact of diagnostic delays on CRC survival, varying by age and cancer stage. Faecal immunochemical testing (FIT) triage could mitigate some effects.	Moderate
Man et al., 2023 [43]	Briefly mentions lung cancer	Clinical Statement / Narrative Review	N/A (review of existing literature)	Age, gender, SES	Urban vs. rural, regional variations	Lower SES and older age negatively influence referral to pulmonary rehabilitation. Women less likely to be referred. Rural and remote areas face challenges in accessing specialised services, exacerbated during pandemic.	Low
Mandrik et al., 2022 [26]	CRC	Decision modelling study using micro-simulation	Simulated population based on English demographic data	Age, gender, IMD quintiles	England	12-month screening pause led to ~ 900 additional CRC deaths in men and ~ 530 in women over 10 years. 50–59 age group most affected (27.4% reduction in diagnoses). Most deprived areas had higher impact (1645 excess YLL per 100000 vs. 916 in least deprived).	High
Nanton et al., 2023 [44]	Multiple, focus on bladder cancer	Review article with case study	N/A (review article)	Ethnicity, SES, age	UK-wide, rural areas mentioned	30% lower odds for minority ethnic group members participating in trials. Low SES a major barrier to participation. Rural areas face challenges in accessing specialised cancer services. Digital divide affects trial participation.	Moderate
Pavlatou et al., 2022 [45]	Thyroid cancer (5.3% of respondents)	Cross-sectional, questionnaire-based online survey	609 valid responses (32 with thyroid cancer)	Age, gender	UK-wide	Younger age associated with greater negative psychological impact across multiple measures. Female gender associated with greater loneliness. 8.1% reported cancellation of thyroid investigations.	Low
Purden et al., 2023 [46]	Breast cancer	Retrospective analysis of patient travel data	1516 patients (400 eligible for 5-fraction regime)	Not directly reported	South-West Wales, rural-city mix	Patients using public transport face significantly longer travel times compared to car users. 5-fraction regime reduced travel burden. One-third of Wales’s population in rural areas impacts travel times.	Moderate
Rajasekaran et al., 2021 [47]	Bone and soft tissue tumours	Retrospective, observational study	347 patients	Age, gender, American Society of Anesthesiologists (ASA) grade	Eight specialist centres across UK	COVID-19 positive patients were older (mean 63 vs. 53 years), but not statistically significant. No significant gender difference in infection rates. Higher ASA grade associated with increased COVID-19 risk.	Moderate
Round et al., 2024 [28]	All cancers, focus on breast, prostate, lung, bowel	Perspective / commentary article	N/A (review of existing data)	Age, deprivation	UK, focus on England	Deprivation associated with lower rates of screening uptake and poorer cancer outcomes. Significant regional variations in emergency presentation rates (e.g., 17% in Wessex vs. 19% England average). Older patients potentially more affected by digital barriers to healthcare access.	Moderate
Shah et al., 2022 [24]	All cancers	Interrupted time series analysis	32 million people across England, Scotland, and Wales	Age, gender, ethnicity, SES	England, Scotland, and Wales	Hospital admissions dropped more for men, most deprived quintile, and ethnic minorities. Overall drop varied by nation (34.2% England, 20.9% Scotland, 24.7% Wales). Black ethnicity faced largest drop in scheduled admissions (62.9%).	High
Stennett & Tsakos, 2022 [48]	Oral cancer	Commentary / review article	N/A (review of existing data)	SES	England	Higher incidence of oral cancers in deprived and vulnerable groups. Substantial decrease in urgent referrals for suspected oral cancer.	Low
Watt et al., 2022 [49]	All cancers, some site-specific analyses	Population-based cohort study	500,000 patients from CPRD Aurum database; national data for England	SES (IMD quintiles)	England	Largest reduction in urgent cancer referrals (17.6%) and first treatments (15.8%) in most deprived areas. Primary care consultations reduced least in most deprived areas (9.6%).	Moderate
Wen et al., 2024 [50]	Prostate cancer	Retrospective cohort study	32,844 lower layer super output areas (LSOAs) in England	Age, ethnicity, income and education deprivation	All of England, urban/rural	Pre-COVID: 41% lower prostate surgery volume in most income-deprived areas. Post-COVID: Gap slightly narrowed to 40.1%. Education deprivation gap widened from 21.3% to 24.7%. Black patients had 2.3% points higher 30-day emergency readmission.	High
Westrop et al., 2024 [51]	Breast cancer	Mixed-methods study	1.7 million eligible breast screening population in South of England	Ethnicity, SES (IMD)	South of England	Change to open invitations exacerbated screening inequalities. For every 1% increase in white patients, screening uptake increased by 0.82% (vs. 0.48% pre-pandemic). IMD effect on uptake decreased slightly post-change.	Moderate

### COVID-19’s impact on cancer care pathways

The impact of the COVID-19 pandemic on cancer care across the UK was multifaceted, affecting the structure, process, and outcomes of care delivery. Applying Donabedian’s model [22], this section summarises how disruptions were experienced at each level of the care continuum and how these disruptions intersected with existing inequalities.

- **Structure**: The pandemic strained healthcare infrastructure across the UK. Hospitals with access to dedicated COVID-free “cold sites” were able to better sustain cancer services such as diagnostics and elective procedures, although these resources were unevenly distributed [23]. Staffing shortages due to redeployment, illness, or isolation further disrupted service capacity, particularly in areas with high COVID-19 prevalence [37]. Financial pressures and the reallocation of resources to pandemic response efforts compounded existing inequities, especially in regions already operating under constrained budgets [27]. The rapid shift towards digital and remote care models highlighted disparities in digital literacy and access, disproportionately affecting older adults and individuals from more deprived areas [44].
- **Process**: Cancer care processes, including screening, diagnosis, and treatment, were significantly disrupted. Multiple studies reported sharp declines in screening uptake across all major programmes during lockdown periods, with slower recovery in deprived and ethnically diverse areas [32, 51]. For example, urgent cancer referrals dropped significantly during the first wave, with more pronounced reductions in lower-income populations [49]. Diagnostic pathways were also affected, with studies documenting delays in secondary care investigations and changes in prioritisation protocols [39]. Treatment services, particularly surgery and systemic therapies, experienced variable reductions, often depending on regional service configurations and hospital-level responses [30]. The temporary suspension or modification of clinical trials further limited treatment options for some patients [47].
- **Outcomes**: While direct evidence on long-term outcomes is still emerging, early findings indicate concerning trends. Modelling studies estimated substantial excess mortality and YLL due to diagnostic and treatment delays, with the burden falling disproportionately on deprived communities [26, 27]. Socioeconomic and ethnic disparities in infection risk among cancer patients further compounded care disruptions and clinical outcomes [11]. Reduced access to timely interventions likely contributed to disease progression in some groups, with consequences expected to manifest over time in survival rates and quality of life [25].

### Key sociodemographic and geographical disparities

The COVID-19 pandemic exposed and exacerbated pre-existing disparities in cancer care across multiple sociodemographic and geographical dimensions. In this review, factors were ranked based on a weighted composite score that combined frequency of identification, consistency of findings across studies, and the magnitude of reported effects. This approach allowed for systematic prioritisation of the most influential determinants. SES, ethnicity, and age consistently ranked highest, demonstrating the strongest and most recurrent associations with disrupted access and adverse outcomes. Gender and regional location, while still relevant, were identified less frequently or with smaller effect sizes. The scoring for each sociodemographic and geographical variable can be found in Additional File 5.

- **SES**: SES consistently emerged as the most influential factor shaping disparities during the pandemic. Individuals from more deprived areas experienced sharper declines in screening uptake, urgent referrals, and access to treatment services [23, 24]. For example, urgent cancer referrals decreased by over 17% in the most deprived quintiles, while the recovery of service use lagged behind less deprived areas [49]. Long-term modelling projected substantially higher excess mortality and YLL in these populations due to delayed diagnoses and treatment [26].
- **Ethnicity**: Ethnic minority groups, particularly Black and Asian patients, were disproportionately affected by disruptions to cancer care. Studies reported significant drops in hospital admissions and screening participation among these populations [11, 24]. In one analysis, breast cancer screening uptake was more strongly associated with White patient populations following the pandemic, indicating a widening ethnic gap [51]. Minority patients were also underrepresented in clinical trials and faced higher COVID-19 infection risks, compounding access and outcome disparities [44].
- **Age**: Disparities by age were evident across various stages of the cancer care pathway. Older adults generally maintained higher screening uptake but faced increased COVID-19-related health risks, which may have influenced decisions around treatment continuation and follow-up care [47]. Conversely, younger and middle-aged adults in deprived areas experienced delayed diagnoses and reduced access to timely interventions [39]. These patterns suggest that age-related disparities were shaped by intersecting socioeconomic and contextual factors.
- **Gender**: Gender differences were also observed, though to a lesser extent. Women maintained higher screening uptake for colorectal cancer throughout the pandemic period [32], while men experienced larger reductions in hospital admissions and treatment access in some settings [24]. Modelling studies further projected greater excess deaths in men due to delayed screening as a result of their lower participation rates [26].
- **Geographical Variation**: Marked regional variation was found in both the scale of service disruption and the speed of recovery. England experienced the largest drop in cancer-related hospital admissions, followed by Wales and Scotland [24]. Within England, more urban and deprived regions experienced greater disruptions to urgent referral pathways and diagnostic capacity [28]. Rural populations, while initially demonstrating higher screening uptake, faced new barriers during the pandemic, including increased travel burden and reduced service availability [46]. These regional inequalities reflect differences in local infrastructure, workforce capacity, and baseline health system resilience.

## Discussion

### Summary of main findings

This review identified clear and consistent evidence that the COVID-19 pandemic exacerbated existing disparities in cancer care across the UK. Socioeconomic deprivation, ethnicity, and age were the most prominent determinants of unequal access and outcomes. These disparities were observed across all stages of the cancer care pathway, with the most pronounced impacts seen in screening participation, diagnostic delays, and reduced treatment activity. Structural challenges such as workforce pressures and uneven access to digital health further compounded these inequalities, disproportionately affecting deprived and minoritised populations. Regional variation also played a significant role, with more deprived and urban areas experiencing deeper service disruptions and slower recovery trajectories. While some services showed signs of adaptation, the cumulative effects of these disruptions are likely to have long-term consequences for cancer outcomes, particularly among already disadvantaged groups.

What is particularly interesting is the strong and consistent influence of SES across multiple strands of the literature. The mechanisms underlying these disparities can be understood through the lens of Fundamental Cause Theory, which posits that social and economic resources shape individuals’ ability to protect their health and secure timely care [52]. During the pandemic, those with greater financial stability, digital access, health literacy, and stronger social networks were better positioned to navigate disrupted pathways, adapt to new modes of care delivery, and seek alternative sources of support. Conversely, individuals in more deprived circumstances had fewer means to compensate for reduced service availability or shifting models of delivery, leaving them more vulnerable to delayed diagnosis and treatment. This theoretical framing helps explain why the pandemic not only reproduced but intensified existing inequalities in cancer care. This systematic review adds to the evidence base by demonstrating how these mechanisms operated across the UK cancer care pathway and by identifying the population groups most affected.

### Implications for policy and practice

The review highlights several issues – the pandemic has brought into sharp focus the need for a health system that is not only resilient but also responsive to existing and emerging inequalities. The evidence reviewed highlights how disruptions to cancer care disproportionately affected those already experiencing disadvantage, particularly individuals from deprived areas, ethnic minority communities, and regions with limited healthcare infrastructure. To mitigate these effects and strengthen future system resilience, a set of integrated policy responses is required.

Structural adaptations such as COVID-free “cold sites” enabled the continuation of diagnostic and treatment services during peak disruption, yet access to such facilities was uneven and must be expanded across all regions to ensure consistency in future crisis preparedness [23]. It also seems addressing inequalities in access requires improving outreach through the deployment of mobile screening units and offering practical support such as transport subsidies and extended clinic hours to facilitate engagement among underserved communities [28]. Community-based education initiatives, developed in partnership with local organisations, can enhance screening participation and early diagnosis, particularly among deprived populations. Ethnic disparities in screening uptake and hospital admissions further point to the need for culturally appropriate public health messaging, linguistically accessible services, and greater diversity in the healthcare workforce. Structural interventions must also include routine cultural competence training for healthcare providers to improve trust and reduce barriers among ethnically minoritised groups [24, 44, 51].

The digital transformation of care, including the rapid expansion of telemedicine, offered opportunities for service continuity but also introduced new access barriers, particularly for older adults, individuals on lower incomes, and those with limited digital literacy [37]. Future service models must address these inequities through comprehensive digital inclusion strategies, including training, access to devices, and non-digital alternatives for those unable to engage remotely. Equity impact assessments should accompany all service innovations to ensure that new models of care do not inadvertently exacerbate disparities [36, 37]. Similarly, treatment innovations such as the use of hypo-fractionated radiotherapy for breast cancer helped reduce patient burden and maintain treatment delivery during the pandemic, although the extent to which these benefits were experienced consistently remains yet to be evaluated. For patients reliant on public transport, particularly in rural areas, travel burdens persisted and may have compromised access [46]. Service redesign efforts must therefore consider the interaction between treatment adaptations and existing social or spatial barriers to ensure equitable access and adherence.

At the system level, the application of risk-stratified prioritisation was critical to protecting vulnerable populations during service reconfiguration and should be retained as a core feature of future contingency planning [24, 42]. However, these efforts must be underpinned by robust data systems capable of disaggregating metrics by SES, ethnicity, gender, and geography to allow for real-time monitoring of disparities. Regions facing a “double hit” from pandemic-related disruption and structural deprivation require targeted investment in infrastructure, workforce development, and service capacity to prevent further entrenchment of inequalities [42]. As mandated by the NHS Constitution, reducing health inequalities is a statutory obligation that must inform the design of integrated care pathways and guide resource allocation decisions [53]. Equity impact assessments should be built into all levels of decision-making to ensure that the recovery and reform of cancer services result in a more inclusive, resilient, and just healthcare system. Without such targeted and sustained action, the structural disparities exposed during the pandemic risk becoming further embedded in the post-COVID landscape.

### Limitations and suggestions for future research

While this review provides a comprehensive synthesis of the impact of COVID-19 on cancer care disparities in the UK, certain limitations should be acknowledged. The rapid evolution of the pandemic meant that many included studies focused on its immediate or short-term effects, with limited data available on long-term outcomes such as survival, recurrence, and quality of life. The predominance of observational designs and heterogeneity in outcome measures made direct comparisons difficult, precluding meta-analysis. Variation in the reporting of sociodemographic variables across studies also presented challenges for uniform interpretation, and there remains a risk of publication bias, with studies demonstrating significant findings more likely to be published. Nonetheless, the use of a structured appraisal framework, inclusion of diverse study types, and UK-wide scope strengthens the reliability and relevance of the findings.

Furthermore, there was only a small number of studies focused specifically on marginalised groups, rural communities, and the operational resilience of cancer pathways, which limited understanding of system vulnerabilities during the pandemic. Some potentially informative studies were excluded because they lacked stratified analyses or UK-specific data, underscoring the need for future work explicitly designed to capture inequalities and system resilience during public health emergencies. Future research should prioritise longitudinal studies that assess the enduring impacts of pandemic-related disruptions on cancer outcomes across different population groups. There is also a need for more rigorous evaluations of interventions introduced during the pandemic, such as telemedicine, hypo-fractionated treatment protocols, and COVID-free care pathways, particularly in relation to their equity impacts. Further work is warranted to explore the effectiveness of culturally tailored outreach and digital inclusion initiatives, especially in ethnically diverse and socioeconomically disadvantaged communities. Additionally, more granular regional analysis is needed to understand how healthcare infrastructure, policy decisions, and population characteristics shaped the resilience of cancer services across different areas of the UK. Mixed-methods research could provide valuable insights into the mechanisms underpinning disparities, capturing both quantitative patterns and patient perspectives. Finally, investment in data infrastructure to support the routine collection of disaggregated and linked health data will be critical for tracking disparities over time and informing equity-oriented health system reform.

## Conclusions

This systematic review provides robust evidence that the COVID-19 pandemic has exacerbated pre-existing disparities in cancer care across the UK. Socioeconomic deprivation, ethnicity, age, and geographical location were consistently associated with disproportionate disruptions to screening, diagnosis, treatment, and outcomes. These inequalities were observed across structural, procedural, and outcome dimensions of the cancer care pathway, reflecting both the direct impacts of the pandemic and the vulnerabilities embedded within the health system. Addressing these disparities requires a sustained policy commitment to equity, informed by data-driven insights and supported by inclusive service models. The pandemic has highlighted opportunities for innovation, such as COVID-free care sites, risk-stratified prioritisation, and accelerated treatment pathways, but their equitable implementation remains essential. As the health system transitions into recovery and reform, targeted investment, ongoing monitoring, and equity-focused planning will be critical to ensuring that lessons from the pandemic translate into a more just and resilient cancer care landscape.

## Supplementary Information

Below is the link to the electronic supplementary material.

Supplementary Material 1

Supplementary Material 2

Supplementary Material 3

Supplementary Material 4

Supplementary Material 5

## Data Availability

The datasets used and/or analysed during the current study are available from the corresponding author on reasonable request.

## Abbreviations

ASA: American Society of Anesthesiologists
BMI: Body Mass Index
CEV: Clinically Extremely Vulnerable
CRC: Colorectal Cancer
DTT: Delays To Treatment
FIT: Faecal immunochemical testing (FIT)
HCC: Hepatocellular Carcinoma
HPV: Human Papillomavirus
IMD: Index of Multiple Deprivation
LSOAs: Lower Layer Super Output Areas
MeSH: Medical Subject Headings
NCA: North of Scotland Cancer Alliance
NHS: National Health Service
NIHR: National Institute for Health and Care Research
PICO: Population, Intervention, Comparison, and Outcomes
SCAN: South East Cancer Network
SES: Socioeconomic Status
UK: United Kingdom
WoSCAN: West of Scotland Cancer Network
YLL: Years of Life Lost
