# Supplementary material for: COVID-19’s disruptions to cancer care pathways and widening of health inequalities in the UK: a systematic review
Source: BMC Health Serv Res. 2026 Mar 26;26:405. doi: 10.1186/s12913-026-14313-8 (PMC13023178; doi:10.1186/s12913-026-14313-8)
Supplement: Supplementary file 1 — Supplementary Material 1 [file 12913_2026_14313_MOESM1_ESM.docx]

**Additional file 1: Search Terms and Results for PubMed, Scopus, and CINAHL**

| **Database** | **Search Terms** | **No. of papers** |
| --- | --- | --- |
| **PubMed** | (Neoplasms[MeSH] OR cancer*[Title/Abstract] OR oncolog*[Title/Abstract] OR malignan*[Title/Abstract])  AND  (COVID-19[MeSH] OR SARS-CoV-2[MeSH] OR coronavirus[Title/Abstract] OR pandemic[Title/Abstract])  AND  (inequalit* OR inequit* OR disparit* OR equity OR equitable OR socioeconomic OR access* OR “Healthcare Disparities”[MeSH])  AND  (“United Kingdom”[MeSH] OR UK[Title/Abstract] OR Britain[Title/Abstract] OR England[Title/Abstract] OR Scotland[Title/Abstract] OR Wales[Title/Abstract] OR NHS[Title/Abstract])  AND  (“2020/01/01”[Date - Publication] : “3000”[Date - Publication]) | 165 |
| **Scopus** | TITLE-ABS-KEY ((neoplasm* OR cancer* OR oncolog* OR malignan*)  AND  (covid-19 OR sars-cov-2 OR coronavirus OR pandemic)  AND  (inequalit* OR inequit* OR disparit* OR equity OR equitable OR socioeconomic OR access* OR “Healthcare Disparities”)  AND  (“United Kingdom” OR uk OR britain OR england OR scotland OR wales OR nhs))  AND  PUBYEAR > 2019 | 279 |
| **CINAHL** | ((MH “Neoplasms+”) OR TI (cancer* OR oncolog* OR malignan* OR neoplasm*) OR AB (cancer* OR oncolog* OR malignan* OR neoplasm*))  AND  ((MH “COVID-19”) OR (MH “SARS-CoV-2”) OR TI (coronavirus OR pandemic) OR AB (coronavirus OR pandemic))  AND  ((MH “Healthcare Disparities”) OR TI (inequalit* OR inequit* OR disparit* OR equity OR equitable OR socioeconomic OR access*) OR AB (inequalit* OR inequit* OR disparit* OR equity OR equitable OR socioeconomic OR access*))  AND  ((MH “United Kingdom+”) OR TI (UK OR Britain OR England OR Scotland OR Wales OR NHS) OR AB (UK OR Britain OR England OR Scotland OR Wales OR NHS))  AND  EM 2020- | 13 |
| **Total number of papers (removing duplicates):** | | **457** |
