# Supplementary material for: COVID-19’s disruptions to cancer care pathways and widening of health inequalities in the UK: a systematic review
Source: BMC Health Serv Res. 2026 Mar 26;26:405. doi: 10.1186/s12913-026-14313-8 (PMC13023178; doi:10.1186/s12913-026-14313-8)
Supplement: Supplementary file 2 — Supplementary Material 2 [file 12913_2026_14313_MOESM2_ESM.docx]

**Additional file 2: Full List of Included Papers in this Systematic Review**

Below is a comprehensive list of the 30 papers selected for inclusion in the systematic review, along with their full citations arranged in alphabetical order.

| **Paper No.** | **Citation** |
| --- | --- |
| **1** | Aggarwal, A., Choudhury, A., Fearnhead, N., Kearns, P., Kirby, A., Lawler, M., ... & Sullivan, R. (2024). The future of cancer care in the UK—time for a radical and sustainable National Cancer Plan. *The Lancet Oncology*, *25*(1), e6-e17. |
| **2** | Ali, J. K., & Riches, J. C. (2021). The impact of the COVID-19 pandemic on oncology care and clinical trials. *Cancers*, *13*(23), 5924. |
| **3** | Baxter, M. A., Murphy, J., Cameron, D., Jordan, J., Crearie, C., Lilley, C., ... & Petty, R. D. (2021). The impact of COVID-19 on systemic anticancer treatment delivery in Scotland. *British Journal of Cancer*, *124*(8), 1353-1356. |
| **4** | Boyle, J. M., Kuryba, A., Blake, H. A., Aggarwal, A., van der Meulen, J., Walker, K., ... & Fearnhead, N. (2021). The impact of the first peak of the COVID‐19 pandemic on colorectal cancer services in England and Wales: a national survey. *Colorectal Disease*, *23*(7), 1733-1744. |
| **5** | Boyle, J. M., Kuryba, A., Blake, H. A., van Der Meulen, J., Fearnhead, N. S., Braun, M. S., & Walker, K. (2024). Inequalities in the recovery of colorectal cancer services during the COVID‐19 pandemic: a national population‐based study. *Colorectal Disease*, *26*(3), 486-496. |
| **6** | Bright, D., Hillier, S., Song, J., Huws, D. W., Greene, G., Hodgson, K., ... & Gjini, A. (2023). Inequalities in colorectal cancer screening uptake in Wales: an examination of the impact of the temporary suspension of the screening programme during the COVID-19 pandemic. *BMC public health*, *23*(1), 546. |
| **7** | Castanon, A., Rebolj, M., Pesola, F., & Sasieni, P. (2021). Recovery strategies following COVID-19 disruption to cervical cancer screening and their impact on excess diagnoses. *British journal of cancer*, *124*(8), 1361-1365. |
| **8** | Creavin, A., Creavin, S., Kenward, C., Sterne, J., & Williams, J. (2023). Inequality in uptake of bowel cancer screening by deprivation, ethnicity and smoking status: cross-sectional study in 86 850 citizens. *Journal of Public Health*, *45*(4), 904-911. |
| **9** | De Souza, S., Kahol de Jong, J., Perone, Y., Shetty, S., Qurashi, M., Vithayathil, M., ... & Sharma, R. (2023). Impact of COVID-19 on 1-Year Survival Outcomes in Hepatocellular Carcinoma: A Multicenter Cohort Study. *Cancers*, *15*(13), 3378. |
| **10** | Dema, E., Sonnenberg, P., Gibbs, J., Conolly, A., Willis, M., Riddell, J., ... & Field, N. (2023). How did the COVID-19 pandemic affect access to condoms, chlamydia and HIV testing, and cervical cancer screening at a population level in Britain?(Natsal-COVID). *Sexually Transmitted Infections*, *99*(4), 261-267. |
| **11** | Green, M. A., McKee, M., Hamilton, O. K., Shaw, R. J., Macleod, J., Boyd, A., & Katikireddi, S. V. (2023). Associations between self-reported healthcare disruption due to covid-19 and avoidable hospital admission: evidence from seven linked longitudinal studies for England. *bmj*, *382*. |
| **12** | Greene, G. J., Thomson, C. S., Donnelly, D., Chung, D., Bhatti, L., Gavin, A. T., ... & Morrison, D. S. (2023). Whole-population trends in pathology-confirmed cancer incidence in Northern Ireland, Scotland and Wales during the SARS-CoV-2 pandemic: a retrospective observational study. *Cancer Epidemiology*, *84*, 102367. |
| **13** | Hamilton, A. C., Donnelly, D. W., Loughrey, M. B., Turkington, R. C., Fox, C., Fitzpatrick, D., ... & Coleman, H. G. (2021). Inequalities in the decline and recovery of pathological cancer diagnoses during the first six months of the COVID-19 pandemic: a population-based study. *British Journal of Cancer*, *125*(6), 798-805. |
| **14** | Hull, S., Williams, C., Basnett, I., & Ashman, N. (2021). Health inequalities worsen with the drop in hospital referrals. *Journal of the Royal Society of Medicine*, *114*(4), 158-159. |
| **15** | Jiwa, N., Takats, Z., Leff, D. R., & Sutton, C. (2021). Breast health screening: a UK-wide questionnaire. *BMJ Nutrition, Prevention & Health*, *4*(1), 206. |
| **16** | Kontopantelis, E., Mamas, M. A., Webb, R. T., Castro, A., Rutter, M. K., Gale, C. P., ... & Doran, T. (2022). Excess years of life lost to COVID-19 and other causes of death by sex, neighbourhood deprivation, and region in England and Wales during 2020: A registry-based study. *PLoS Medicine*, *19*(2), e1003904. |
| **17** | Lee, S. F., Nikšić, M., Rachet, B., Sanchez, M. J., & Luque-Fernandez, M. A. (2021). Socioeconomic inequalities and ethnicity are associated with a positive COVID-19 test among cancer patients in the UK Biobank cohort. *Cancers*, *13*(7), 1514. |
| **18** | Loveday, C., Sud, A., Jones, M. E., Broggio, J., Scott, S., Gronthound, F., ... & Turnbull, C. (2021). Prioritisation by FIT to mitigate the impact of delays in the 2-week wait colorectal cancer referral pathway during the COVID-19 pandemic: a UK modelling study. *Gut*, *70*(6), 1053-1060. |
| **19** | Man, W., Chaplin, E., Daynes, E., Drummond, A., Evans, R. A., Greening, N. J., ... & Singh, S. J. (2023). British thoracic society clinical statement on pulmonary rehabilitation. *Thorax*, *78*(Suppl 5), s2-s15. |
| **20** | Mandrik, O., Chilcott, J., & Thomas, C. (2022). Modelling the impact of the coronavirus pandemic on bowel cancer screening outcomes in England: a decision analysis to prepare for future screening disruption. *Preventive Medicine*, *160*, 107076. |
| **21** | Nanton, V., Bryan, R. T., Pope, A. M., Hughes, A., Jefferson, K., Catto, J. W., ... & James, N. D. (2023). Boosting and broadening recruitment to UK cancer trials: towards a blueprint for action. *BMJ Oncology*. |
| **22** | Pavlatou, M. G., Žarković, M., Hegedüs, L., Priestley, J., McMullan, C., & Perros, P. (2022). A survey on the psychological impact and access to health care of thyroid patients during the first SARS‐COV‐2 lockdown. *Clinical Endocrinology*, *96*(6), 869-877. |
| **23** | Purden, J., Jackson, T., Tales, A., & Lewis, R. (2023). Access and travel burden associated with breast radiotherapy attendance pre-and post-COVID-19 pandemic. *Journal of Radiotherapy in Practice*, *22*, e114. |
| **24** | Rajasekaran, R. B., Ashford, R. U., Cosker, T. D., Stevenson, J. D., Jeys, L., Pollock, R., ... & Carr, A. (2021). What proportion of patients with bone and soft tissue tumors contracted Coronavirus-19 and died from surgical procedures during the initial period of the COVID-19 pandemic? Results from the multicenter British Orthopaedic Oncology Society Observational Study. *Clinical Orthopaedics and Related Research®*, *479*(5), 1158-1166. |
| **25** | Round, T., Sethuraman, L., Ashworth, M., & Purushotham, A. (2024). Transforming post pandemic cancer services. *British Journal of Cancer*, *130*(8), 1233-1238. |
| **26** | Shah, S. A., Brophy, S., Kennedy, J., Fisher, L., Walker, A., Mackenna, B., ... & Katikireddi, S. V. (2022). Impact of first UK COVID-19 lockdown on hospital admissions: Interrupted time series study of 32 million people. *EClinicalMedicine*, *49*. |
| **27** | Stennett, M., & Tsakos, G. (2022). The impact of the COVID-19 pandemic on oral health inequalities and access to oral healthcare in England. *British dental journal*, *232*(2), 109-114. |
| **28** | Watt, T., Sullivan, R., & Aggarwal, A. (2022). Primary care and cancer: an analysis of the impact and inequalities of the COVID-19 pandemic on patient pathways. *BMJ open*, *12*(3), e059374. |
| **29** | Wen, J., Santos, R., Siciliani, L., & Proctor, A. (2024). Socioeconomic inequalities in hospital access for prostate cancer before and after COVID-19. *Socio-Economic Planning Sciences*, *94*, 101914. |
| **30** | Westrop, S. J., Thomas, A., Williams, A., Johnson, F., Liao, H., Edlin, K., ... & Maclean, R. (2024). Impact of changes to invite methodology on equality of access to the National Breast Screening Programme in the South of England. *Journal of Medical Screening*, *31*(2), 115-118. |
