# Supplementary material for: COVID-19’s disruptions to cancer care pathways and widening of health inequalities in the UK: a systematic review
Source: BMC Health Serv Res. 2026 Mar 26;26:405. doi: 10.1186/s12913-026-14313-8 (PMC13023178; doi:10.1186/s12913-026-14313-8)
Supplement: Supplementary file 3 — Supplementary Material 3 [file 12913_2026_14313_MOESM3_ESM.docx]

**Additional file 3: Custom Data Extraction Form**

This section is designed to systematically extract relevant information from each included study. It covers key aspects of the study design, population characteristics, geographical context, COVID-19 related information, cancer care aspects, disparity measures, and main findings. Please complete all applicable fields based on the information provided in the study.

| **1. Study Identification** | |
| --- | --- |
| Title |  |
| Author(s) |  |
| Year of publication |  |
| Journal |  |
| DOI/URL |  |
| **2. Study Characteristics** | |
| Study design |  |
| Sample size |  |
| Sampling method |  |
| Study duration |  |
| Analysis method |  |
| **3. Population Characteristics** | |
| Cancer type(s) |  |
| Age range and distribution |  |
| Gender distribution |  |
| Ethnicity breakdown |  |
| Socioeconomic indicators |  |
| **4. Geographical Context** | |
| Specific regions/areas |  |
| Urban/rural classification |  |
| Healthcare system context |  |
| **5. COVID-19 Related Information** | |
| Specific pandemic time period studied |  |
| Waves or lockdown periods mentioned |  |
| Pandemic-related policies |  |
| **6. Cancer Care Aspects Examined** | |
| Screening programmes affected |  |
| Diagnostic services impacted |  |
| Treatment modalities studied |  |
| Follow-up care |  |
| **7. Disparity Measures** | |
| Specific outcomes used to measure disparities |  |
| How disparities were quantified/described |  |
| Baseline (pre-pandemic) disparity levels, if reported |  |
| **8. Key Findings** | |
| Key sociodemographic factors (with key statistics and quoted/estimated effect sizes where applicable) |  |
| Key geographic factors (with key statistics and quoted/estimated effect sizes where applicable) |  |
| **9. Potential Confounding Factors** | |
| Factors considered |  |
| How they were addressed |  |
| **10. Intervention Details (if applicable)** | |
| Description of interventions to address disparities |  |
| Duration and intensity of interventions |  |
| **11. Limitations** | |
| Limitations identified by the authors |  |
| Limitations identified by the reviewer |  |

Completed by: _________________ Date: _________________

Second reviewer check: _________________ Date: _________________

Discrepancies resolved: □ Yes □ No Date: _________________
