# Supplementary material for: COVID-19’s disruptions to cancer care pathways and widening of health inequalities in the UK: a systematic review
Source: BMC Health Serv Res. 2026 Mar 26;26:405. doi: 10.1186/s12913-026-14313-8 (PMC13023178; doi:10.1186/s12913-026-14313-8)
Supplement: Supplementary file 4 — Supplementary Material 4 [file 12913_2026_14313_MOESM4_ESM.docx]

**Additional file 4: Scoring System Overview and Study Appraisal Form**

This section aims to evaluate the quality, relevance, and significance of the study using a bespoke scoring system. Each domain is scored on a point scale as indicated. The total score (maximum 13 points) determines the overall importance category of the study. Please provide a justification for each score assigned.

Scoring System Overview:

- Methodological Rigour: 0-3 points (0 = major flaws, 3 = high quality)
- Relevance to Research Question: 0-3 points (0 = not relevant, 3 = highly relevant)
- Scope of Cancer Care Coverage: 0-2 points (0 = very narrow, 2 = comprehensive)
- Sample Coverage: 0-2 points (0 = limited/unclear, 2 = multiple regions/large population)
- Effect Size and Significance: 0-3 points (0 = no clear effect, 3 = large and significant effect)

Total Score Interpretation:

- High importance: 11-13 points
- Moderate importance: 7-10 points
- Low importance: 0-6 points

| **1. Methodological Rigour (0-3 points)** | |
| --- | --- |
| Score |  |
| Justification |  |
| **2. Relevance to Research Question (0-3 points)** | |
| Score |  |
| Justification |  |
| **3. Scope of Cancer Care Coverage (0-2 points)** | |
| Score |  |
| Justification |  |
| **4. Sample Coverage (0-2 points)** | |
| Score |  |
| Justification |  |
| **5. Effect Size and Significance (0-3 points)** | |
| Score |  |
| Justification |  |
| **6. Overall Score and Importance Category** | |
| Total Score: ___ / 13  Importance Category: □ High (11-13) □ Moderate (7-10) □ Low (0-6) | |
| **7. Additional Notes** | |
| Any other relevant information or reviewer comments: | |

Completed by: _________________ Date: _________________

Second reviewer check: _________________ Date: _________________

Discrepancies resolved: □ Yes □ No Date: _________________
