# Supplementary material for: COVID-19’s disruptions to cancer care pathways and widening of health inequalities in the UK: a systematic review
Source: BMC Health Serv Res. 2026 Mar 26;26:405. doi: 10.1186/s12913-026-14313-8 (PMC13023178; doi:10.1186/s12913-026-14313-8)
Supplement: Supplementary file 5 — Supplementary Material 5 [file 12913_2026_14313_MOESM5_ESM.docx]

**Additional File 5: Scoring for Each Sociodemographic and Geographical Variable**

To identify the most significant sociodemographic and geographical factors influencing cancer care disparities during the COVID-19 pandemic, factors were ranked based on a composite score derived from:

1. **Frequency**: Number of high and moderate importance studies that identified the factor as significant.
2. **Consistency**: Degree of agreement across studies regarding the factor’s impact.
3. **Effect** **Magnitude**: Overall strength of the factor’s effect across studies.

These components were combined into a weighted score for each factor, calculated as:

**(Number of high importance studies × 2 + Number of moderate importance studies) × (Consistency score: 1-3) × (Effect Magnitude score: 1-3)**

The top three factors with the highest composite scores were designated as key factors for discussion in the synthesis and subsequent regression analyses.

Summary of scores for each of the sociodemographic and geographical factors

| **Factors** | **Frequency** | | **Consistency Score** | **Effect Magnitude score** | **Total Score** |
| --- | --- | --- | --- | --- | --- |
|  | **High** | **Moderate** |  |  |  |
| Socioeconomic Status | 5 | 8 | 3 | 3 | 162 |
| Ethnicity | 2 | 7 | 3 | 3 | 99 |
| Age | 3 | 9 | 2 | 3 | 90 |
| Gender | 3 | 3 | 2 | 2 | 36 |
| Location | 1 | 7 | 2 | 2 | 36 |
